# Compassionate collaborative care: an integrative review of quality indicators in end-of-life care

**DOI:** 10.1186/s12904-017-0246-4

**Published:** 2017-12-01

**Authors:** Kathryn Pfaff, Adelais Markaki

**Affiliations:** 1 Faculty of Nursing, University of Windsor, Rm. 312 Toldo Health Education Centre, 401 Sunset, Windsor, ON N9B 3P4 Canada; 2 School of Nursing, University of Alabama at Birmingham, 1720 2nd Ave. South, Birmingham, AL 35294-1210 USA

**Keywords:** Compassion, Collaboration, Interprofessional relations, Empathy, Patient-centered care, Palliative care, End-of- life care, Organizational models, Quality indicators

## Abstract

**Background:**

Compassion and collaborative practice are individually associated with high quality healthcare. When combined in a compassionate collaborative care (CCC) practice framework, they are reported to improve health, strengthen care provision, and control health costs. Little is known about how to integrate and measure CCC, yet it is fundamentally applied in palliative and end-of-life care settings. This study aimed to identify quality indicators of CCC by systematically reviewing and synthesizing the current state of the palliative and end-of-life care literature.

**Methods:**

An integrative review of the palliative and end-of-life care literature was conducted using Whittemore and Knafl’s method. Donabedian’s healthcare quality framework was applied in the data analysis phase to organize and display the data. The analysis involved an iterative process that applied a constant comparative method.

**Results:**

The final literature sample included 25 articles. Patient and family-centered care emerged as a primary structure for CCC, with overarching values including empathy, sharing, respect, and partnership. The analysis revealed communication, shared decision-making, and goal setting as overarching processes for achieving CCC at end-of-life. Patient and family satisfaction, enhanced teamwork, decreased staff burnout, and organizational satisfaction are exemplars of outcomes that suggest high quality CCC. Specific quality indicators at the individual, team and organizational levels are reported with supporting exemplar data.

**Conclusions:**

CCC is inextricably linked to the inherent values, needs and expectations of patients, families and healthcare providers. Compassion and collaboration must be enacted and harmonized to fully operationalize and sustain patient and family-centered care in palliative and end-of-life practice settings. Towards that direction, the quality indicators that emerged from this integrative review provide a two-fold application in palliative and end-of-life care. First, to evaluate the existing structures, processes, and outcomes at the patient-family, provider, team, and organizational levels. Second, to guide the planning and implementation of team and organizational changes that improve the quality delivery of CCC.

## Background

Since the early 2000s, there has been generalized concern over the decreasing state of compassion in health systems across developed countries [1, 2]. Defined as “the recognition, empathic understanding of and emotional resonance with the concerns, pain, distress or suffering of others coupled with motivation and relational action to ameliorate these conditions” [3]. Not only is it viewed as a guiding foundation for ethical practice among healthcare professionals and organizations, but also as a cornerstone of quality healthcare by patients, families, clinicians, and policy makers [4–7]. Emerging evidence shows a relationship among compassionate care, improved patient outcomes and enhanced provider well-being [6, 7]. Despite efforts, compassion remains elusive in many organizations and care settings, and is poorly conceptualized [8] and empirically understood [6]. According to a recent scoping review of the compassion healthcare literature, there is a lack of patient and family data to inform the body of literature [6]. Looking beyond patient and family perspectives and into the team and organization is further required to understand their influence on values and practices [9].

Collaborative practice has numerous definitions, but the majority agree that it involves multiple disciplines of healthcare team members who work with patients and families to achieve common goals through processes, such as shared communication and decision-making [10–12]. It is a practice model whose core domain involves a patient and family-centred approach [11]. Collaborative practice has been shown to improve health outcomes in and across care sectors and settings [12, 13], and is linked with higher accessibility to care, better chronic disease management, patient safety, and healthy workplaces [11–13]. Despite a growing body of literature, the integration of collaborative practice continues to lag behind in many healthcare settings [14].

As an exception, palliative and end-of-life care settings are places where compassionate patient and family centered care is the priority of the interprofessional (IP) team. This led us to theorize that compassion is the lever or ‘missing antecedent’ for fully operationalizing and sustaining collaborative practice in end-of-life care settings [15]. Compassion is a foundational value underlying the modern hospice movement [16–18], and a core concept of palliative care. It involves a holistic approach in which IP care providers support patients and families throughout diagnosis, disease stages, death and bereavement [19]. Compassion is also considered a marker of spiritual care, a facilitator for ameliorating existential suffering towards end-of-life [6], and an enabler of an integrated patient-centered approach [20]. Nevertheless, there is no robust evidence that describes how to systematically promote and improve the quality of compassionate collaborative care (CCC) in palliative or hospice care settings.

In 2014, the Schwartz Center for Compassionate Healthcare and the Arnold P. Gold Foundation convened an expert panel to recommend timely steps for integrating compassion and collaboration [3]. Panel members included patients, family members, advocates, clinicians, health profession educators, licensure and accreditation agency representatives, funders, and administrators. The Compassionate Collaborative Care Model and Framework was identified as a vehicle for improving health and experiences of care while controlling health-related costs [3]. Making CCC the standard of care in every healthcare organization and patient encounter was agreed upon as the ultimate vision for excellence in healthcare [3, 21]. Although the report identifies the major attributes and provider skills associated with CCC, it provides few steps for its assimilation into healthcare teams, settings, and organizations. Therefore, greater understanding of organizational culture and system change processes is essential [3]. Without this knowledge, teams and organizations will remain continually challenged to integrate and measure the impact of compassionate collaborative care.

Measuring the quality of care and services through indicators, including patient and family satisfaction, has become increasingly important. According to Schuster and colleagues, key indicators can be measures of structure, process, and outcome, classified according to type of care, function, and modality [22]. For certain conditions, treatments or patient populations, indicators without evidence, based solely on professional consensus, may be all that is feasible [23]. Because growing evidence suggests that practicing with compassion leads to better outcomes [6, 7, 24, 25], it is important to understand the nature of CCC and its quality indicators. As CCC is philosophically and fundamentally applied in palliative and end-of-life care, this body of literature is theoretically appropriate for examination.

### Aim

The aim of this study was to identify quality indicators of CCC by systematically reviewing and synthesizing the current state of the palliative and end-of-life care literature.

## Methods

Whittemore and Knafl’s methodology was chosen given its ability to synthesize literature from a wide range of sources [26]. It involves five phases: problem identification, literature search, data evaluation, data analysis and presentation. Donabedian’s healthcare quality framework [27, 28], as adopted by Mainz [23], was used to guide the data analysis phase. A conceptual definition of CCC was created to focus the review. It was based on the WHO Framework for Action on Interprofessional Education & Collaborative Practice [12] and the Compassionate Collaborative Care Model and Framework [3] as follows:

*Compassionate collaborative care (CCC) is a process through which caregivers from different professional and non-professional backgrounds work together with patients and families to deliver care that recognizes, understands and responds to concerns, pain, distress, or suffering, with the aim to promote positive patient-family, team, and organizational outcomes across healthcare settings.*

### Literature search

The following online databases were searched for relevant key terms: Medline, CINAHL, ProQuest, and PubMed. Numerous search terms were used in various combinations. These terms were identified from a preliminary review of the literature and author expertise, and included the following algorithm: (interprofessional OR interdisciplinary OR multidisciplinary OR transdisciplinary) AND (collaboration OR cooperation OR practice OR team work OR teamwork OR care OR caring) AND (compassion or empathy or sympathy) AND (hospice OR palliative OR end-of-life OR end of life). Truncation and wildcard symbols were applied to maximize retrieval of related reports.

Inclusion criteria were as follows: peer-reviewed, published in English, original research, systematic review, literature review, case study, conference proceedings, or position statements. The settings of interest were acute care, hospice palliative care, and long-term care. Given the conceptual nature of the review, there was no limit on publication date. We excluded studies that did not meet the inclusion criteria reported above. Studies conducted in home or community settings were also excluded given the heterogeneity in their structures and processes.

**Fig. 1:**
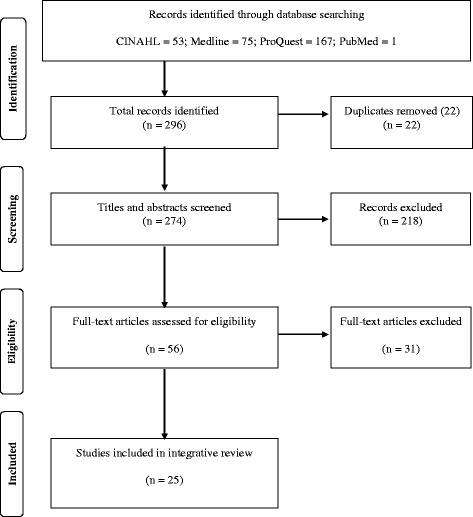
Literature Search and Inclusion

The literature search produced a total of 296 citations. The removal of 22 duplicates left 274 citations for title and abstract screening. Two hundred and eighteen articles were rejected during title and abstract screening. This number included one article (dated 1987) that was not accessible from library and digital sources. The title and abstract screening process produced 56 articles that were eligible for full manuscript screening. During the full manuscript review phase, 31 articles were rejected, resulting in a final literature sample of 25 articles. Outcomes of the literature search and screening procedures are reported in Fig. 1.

### Data evaluation

Both authors independently screened each title and abstract and documented their recommendation to include or exclude. When screening the titles and abstracts of each citation, we maintained a constant focus on the aim of the review and the question: “*Does the article potentially address structures, processes or outcomes of compassionate collaborative care for patients at end-of-life of any age group in acute care (any unit), tertiary care (hospice, palliative care) or long-term care?”* Upon comparing the independent screening results, disagreements were thoroughly discussed until agreement was reached to include or reject. Following this phase, full manuscripts were retrieved and the same strategy was applied for inclusion/exclusion. Articles that did not meet inclusion criteria were eliminated from literature sample. Characteristics of the literature sample are reported in Table 1 **.**

**Table 1:** Data Abstraction Framework for CCC Indicators

CCC Indicators	Individual Level (patient-family- provider)	Team Level	Organizational Level
Structure			
Attributes and characteristics, the “what and where”, supportive resources (material and human)	Cell 1	Cell 2	Cell 3
Process			
Interventions, what is done in giving and receiving CCC, the “how”, actions, steps, change that occurs over time	Cell 4	Cell 5	Cell 6
Outcome			
Short-term and long-term effects and impacts of CCC on patients, providers, teams, and organizations	Cell 7	Cell 8	Cell 9

Although not required in an integrative review [26], the articles were appraised for quality. We believed this was necessary for users to be able to interpret the usefulness and transferability of the review findings to practice and policy [29]. We used the qualitative and quantitative criteria of Letts et al. [30] and Polit and Beck [31], respectively to assess the rigour of each study. The articles were then ranked on a scale of 1 (weak) to 5 (strong) based on the presence or absence of evidence to support the criteria. Six articles reflected research studies (5 = qualitative; 1 = mixed methods). We retrieved one meta-synthesis of the qualitative literature, and two quality improvement (QI) program evaluations. Sixteen documents reflected non-research articles that included position/consensus statements (*n* = 4), case study reports (*n* = 2), literature reviews (n = 2), and narrative summaries of Schwartz Rounds (*n* = 8). Given the nature of these reports and lack of reliable appraisal tools, these articles did not undergo quality appraisal. Nevertheless, they were considered low forms of evidence [32]. Two of the qualitative studies were scored as moderately-high (score = 4). The remaining articles were scored between 1 and 3. The weak scores reflect studies that lacked comprehensive literature reviews and clear reporting of design and methods. Although there is criticism about inclusion of low quality of studies in systematic reviews, these concerns largely relate to the bias associated with meta-analyses [33]. Given the lack of research in this area, including all studies that met inclusion was necessary to understand this phenomenon.

### Data analysis

**Fig. 2:**
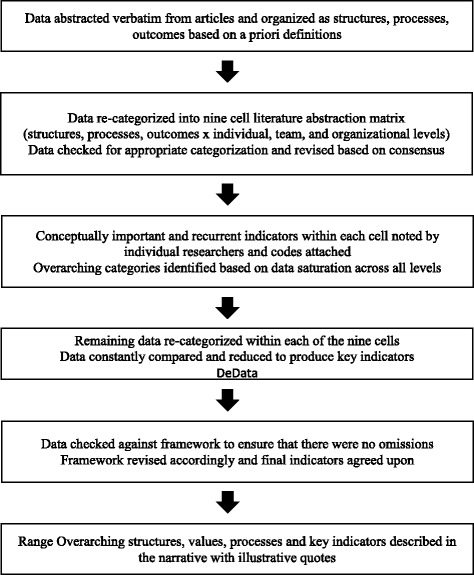
Phase two data analysis

The process by which data analysis and synthesis was conducted is displayed in Fig. 2. It involved an iterative process that applied a constant comparative method [34] throughout data abstraction, reduction, display, conclusion drawing, and verification stages [35]. We continually focused on the research question and the adopted conceptual definition of compassionate collaborative care throughout the analysis. The process involved two phases: (1) literature abstraction and (2) data reduction and display. To achieve consistent coding and categorization of the data, the researchers met weekly to compare and agree on the attribution of the data.

### Literature abstraction

During the literature abstraction phase, data that described the structures, processes, and/or outcomes of CCC were abstracted verbatim to an Excel file to facilitate coding, categorization, and sharing. The following definitions, stemming from Mainz [23], were applied as codes for the quality indicators:

- *‘Structure’* denotes the attributes of settings where care occurs. It refers to health system characteristics that affect the ability to meet the health care needs of individual patients, families, or a community. Structural indicators describe the type and amount of resources used (i.e. staff, clients, money, beds, supplies, buildings) in order to answer whether care is provided under favorable or unfavorable conditions to good care.
- *‘Process’* denotes what is actually done in giving and receiving care. Processes are a series of inter-related activities undertaken to achieve objectives. Process indicators measure the activities and tasks in patient episodes of care. For some researchers, seeking care and carrying it out are also viewed as process indicators.
- *‘Outcome’* describes the effects of care on patient and/or population health status. These may include knowledge improvement, changes in behavior and ultimately, satisfaction with care. Outcome indicators are states of health or events that follow care and should be evidence-based.

### Data reduction and display

The data from phase one were further abstracted into Table 2 for reduction and display across the patient-family-provider, the team, and the organization levels. Numerical codes (1 through 9) were attached to each data point to support the reliability and consistency of the data analysis. As the analysis proceeded, the cell descriptors were refined to best fit the data [36]. The data were further reduced into sub-categories within each of the cells. This also involved a rigorous and iterative process of comparing data points within each cell to all other data in each cell.

**Table 2:** Literature sample and appraisal

Author (Year)	Country	Design	Setting/Sample	Disciplines/Roles	Relevant Findings and Extraction	Appraisal Grade^a^ (1–5)
Addicott (2013) [56]	U.K.	Qualitative study based on 4 case studies Semi-structured interviews	4 high-performing care homes in end-of-life care	Social workers, palliative care specialists, community nurses, care home managers, RNs, care assistants	Three pivotal factors to high-quality EoLC in care homes: advance care planning (ACP), multidisciplinary communication and work, dignified and compassionate care. ACP as a useful trigger for beginning communication with residents and other care professionals. Strong leadership can motivate compassionate care.	2
American Academy of Pediatrics (2013) [37]	U.S.	Policy Statement / Pediatric Palliative Care-Pediatric Hospice Care (PPC-PHC) Guidelines based on published observational studies, expert opinion, and consensus statements.	N/A	N/A	Model Principles: 1. Patient-centered and family engaged 2. Respect & partnering 3. Quality, access & equity 4. Care across age spectrum & life span 5. Integration into the continuum of care 6. Universal preparedness & consultation 7. Research & continuous improvement Teams should have sufficient collective expertise and adequate staff to address child/family needs**.** Organizations should have dedicated interdisciplinary PPC- PPC-PHC teams that facilitate clear, compassionate discussions supporting families and staff beyond EOL period. PPC-PHC involves collaborative, integrated multimodal care (cure seeking, life-prolonging, comfort-enhancing, QOL-enriching)	N/A
Borhani et al. (2013) [38]	Iran	Descriptive exploratory qualitative study	A teaching hospital in Islamic South-East Iran, 12 ICU nurses	Nurses	Commitment to care is expected until the last moment. Nursing challenges include ethical issues, family expectations, religious issues and miracles. Care involves awareness of the needs of dying patients, identifying impending death, promoting comfort and spiritual care, and caring relationships	4
Cook et al. (2015) [39]	Canada	Mixed methods	Dying pts. in Med/Surg ICU, their families and clinicians	Physicians, residents, nurses, social workers, chaplains, family members	Honouring pt. wishes in ICU involved humanizing the environment, personal tributes, family reconnections, rituals & observances, dignifying the patient, giving the family a voice as partners in the caring process instead of “visitors”, fostering clinician compassion by encouraging self-awareness, reflection and sense of collective purpose	2
Costello (2001) [57]	U.K.	Ethnographic study Participant observation, semi-structured interviews	74 pts. in elderly care wards in a large hospital, 29 nurses, 8 physicians	Nurses, physicians	Hospital culture, mores and beliefs impact the experiences of older dying pts. Bracketing out time provision of psychological care.	4
Cox (2004) [40]	U.S.	Review of literature and position statement (Emergency Nurses’ Association, American College of Emergency Physicians, American Trauma Society)	Comprehensive Pediatric Bereavement Programs (CPBP)	Multidisciplinary Bereavement Committee (nurses, physicians, SWs, chaplains, child life specialists)	Key elements of CPBP: team approach, recognition of cultural differences, integration of family into care of the dying, memory packets or boxes, support groups, resource lists and information, remembrance ceremony, continued contact with family, staff education and development, program evaluation and feedback.	N/A
Hanson & Cullihall (1996) [41]	Canada	Care study (for teaching purposes)		Palliative care team: nurse, physician, SW, chaplain, domiciliary care sister	Patient and family holistic care was delivered by an interdisciplinary palliative team. Primary nursing role involved coordination and integration with other health team members continuity of care, provision of comfort care and symptom management. The care model improves quality of life for the dying and enables informed choices for patients and families	N/A
Kayser-Jones et al. (2005) [58]	U.S.	Content analysis	33 residents in a 25-bed hospice unit within a large long-term care facility	Hospice team (certified physician, nurse manager, RNs, LVNs, CNAs, SW, activity therapist, spiritual counsellor, volunteer coordinator)	The hospice environment provides physical-psycho-social-spiritual care as a therapeutic community, and reflects compassion. The hospice team core values include communication at all levels and development of sense of community. There is awareness of how the environment influences care (attention to details, minimum noise, soft background music, common meal area, garden homelike, supportive, family). Alternative, creative approaches to symptom management were used Social and spiritual events (Happy Hour, Memorial Service) provided a sense of community. Needs of resident prioritized over paperwork.	1
Kehoe (2006) [42]	U.S.	Metasynthesis of qualitative studies	65 US hospice nurses (in 5 qualitative studies)	Hospice nurses / hospice team	Strong personal foundations are complemented by firm professional supports. Team includes the patient’s family, nurses, social workers, physicians, and other professionals and volunteers who “cocoon” the dying. Hospice nurses are collaborators, valuing, seeking, and offering support to the IP team. For nurses, is important to develop a sense of the abilities of others on the hospice team.	N/A
Knuti et al. (2003) [43]	U.S.	Non-research Schwartz Rounds – case study focused on caregiver (physician) who becomes pt. (cancer surpriser)	Oncology	Physicians (oncologist), patient (physician), spouse (psychologist), social worker, clinical nurse practitioner, nurse, fellow	Processes involve active planning, ongoing support from relatives, friends and the team as resources described as “circles of strength”, being honoured and cared for by the team; patient and family involvement in decisions; open and direct communication; promoting self-care; proactive and timely referrals; IP mentoring. Outcomes include intimacy, happiness after diagnosis, hope, changing relationships and perspectives, enhanced spirituality.	N/A
Krakauer (2000) [44]	U.S.	Non-research Schwartz Rounds – case study focused on acute palliative care and the way comfort was provided to a young adult in his last days of life (principle of double effect)	Hematology – Oncology Dept. & Palliative Care Service	Facilitator, Ward Nurse, SW, Chaplain, Palliative Care Nurse, Palliative Care Physician, Pt’s family, Pharmacist	Patients expected caregivers to listen to needs and respect wishes. Care involved tremendous integration and teamwork between patient, family, nursing, palliative care, and pharmacy. The team considered the best place for the patient to die taking into account family wellbeing. Having a safety net is important to protect a dying patient from acute suffering. Support from, consultation and collaboration with other providers improved pain management. Family perceived making the right decision and felt like part of the team. Positive outcomes included protocol development for use of barbiturates for intractable suffering. Spiritual peace was achieved for family, friends and staff. Team education is needed to address ethics of the “double effect”.	N/A
Lintz et al. (1999) [45]	U.S.	Non-research Schwartz Rounds – case study focused on aggressive palliative treatment & psychosocial issues faced by pts., families and caregivers	Hematology-Oncology Dept.	Physician, nurse, medical oncologist, psychiatrist, SW	The team worked with the family to proceed through fertility treatments, pregnancy, and birth. Instilling hope and helping patients address difficult questions, and make decisions is a significant part of care. The team works together to decide to continue or cease treatment. Acknowledging the patient’s positive impact on the caregiver’s life.	N/A
Moore & Phillips (2009) [61]	U.S.	Non-research Schwartz Rounds – summarizes findings from an independent study commissioned by Schwartz Center (SC) that examines Rounds’ outcomes, discusses their utility in providing support and offers lessons learned for others who may want to consider Rounds’ implementation	VA Hospital	SW, hospice/palliative medicine physicians	Implementing Schwartz Rounds requires commitment by hospital administration, human resources and formalized planning. Reflecting on the emotional aspect of care enhances caregivers’ ability to deal with a similar situation in the future. Rounds provide support towards empathic practice of medicine (i.e. reflection, self-monitoring, processing emotion and coping with its effects). Unanticipated outcomes: patient-centered changes in institutional policy or practice; greater use of palliative care teams/enhanced palliative care services; improved linkages among hospital services to better meet the needs of veterans with mental health disorders and substance abuse; discussion among staff about advanced illness and palliative care issues.	N/A
Penson et al. (2000) [60]	U.S.	Non-research Schwartz Rounds – case study focused on burnout	Oncology	Physicians, social worker, clinical nurse practitioner, nurse, fellow	Participants emphasized communication, partnership, having the right attitude and the team as being “what saves all of us”. The rounds provide spaces for connecting with the team and learning about the patient as an individual. Reported positive outcomes included reduced burnout and stress, ability to recognize stress and burnout in self and others, improved coping and teamwork and team support.	N/A
Penson et al. (2002a) [46]	U.S.	Non-research Schwartz Rounds – case study focused on bereavement	Oncology	Social worker, oncologist, palliative care nurse, infusion nurse, pediatric oncologist, palliative care physician, psychologist	The team was viewed as an extended family. Caring involves honouring the personal relationship, meeting patient’s needs as survivors, and realizing the need to sacrifice. Processes included: phone calls, bereavement care programs (sending cards, memorial services, saying good-bye to patient, family, friends), bereavement rounds for staff. Family valued receiving phone calls, cards and letters.	N/A
Penson et al. (2002b) [47]	U.S.	Non-research Schwartz Rounds – case study focused on negotiating cancer treatment in adolescents	Oncology	Social worker, ward nurse, oncologist, palliative care nurse specialist, psychiatrist	Discussion centred around several topics: care decisions, sharing prognosis and options, maintaining supporting patient wishes, mobilizing transition to hospice care, saying good-bye, team communication, commitment and working relationships. Positive outcomes were allowing *“different family members to establish their own relationship with team members… members of the team [were able] to share the sadness and the challenges of working with the family and to ‘hold’ the emotion that these cases evoke”.*	N/A
Penson et al. (2005) [48]	U.S.	Non-research Schwartz Rounds – case study focused on fear of death	Oncology	Physician, nurse, chaplain, psychiatrist, oncologist, social worker, palliative care physician	Discussion emphasized the challenges and benefits of addressing conflicts, responding to difficult questions, being empathetic and taking time to listen and be present. Careful care planning alleviated fear and depression. The process resulted in team role fulfillment. [	N/A
Puchalski et al. (2006) [49]	U.S.	Non-research Model for interdisciplinary spiritual care	N/A	Applicable to physician, social worker, nurse, chaplain	The document integrates a “Call to Action”. The model emphasizes adherence to a biopsychosocial-spiritual model of care that is practised by all members of the healthcare team. Self-awareness, ongoing interdisciplinary team communication ensures that patient and family have comprehensive compassionate plan of care. Compassionate presence involves intention, openness, connections with others, and comfort with uncertainty. It is relationship-centred, not agenda-driven. It enriches the work of each healthcare professional richer through collective action.	N/A
Puchalski et al. (2014) [50]	U.S.	Non-research Consensus findings re standards and strategies for integrating spiritual care	N/A		Organizational policies should promote spiritual compassionate care across the organization, at the bedside with patients and families, in staff relationships, and at all levels of leadership. It has potential to transform and heal all parties.	N/A
Rushton et al. (2006) [51]	U.S.	Quality Improvement Evaluation of an IP grief program	Children’s hospital	Physicians, nurses, social workers, child life specialists, bereavement coordinator, family care coordinator, volunteers	The grief program involved all units, departments, disciplines, leaders and volunteers. Timing and training were provided. Change was guided by a hypothesis: “health care professionals will provide better care and support to seriously ill children and their families when they feel supported personally and professionally in their work.” There were four interventions: 1. Compassionate Care Network - integrated palliative and EoLC information and expertise across all units 2. Institutional palliative care rounds 3. Patient care conferences 4. Bereavement debriefing sessions Surveillance data were collected. The team benefited from increased teamwork and morale, knowledge about others’ expertise, knowledge about advocacy, and patient and family care in the terminal phase. Frequency of referrals, conferences and meetings increased.	1
Schermer Sellers (2000) [52]	U.S.	Research evaluation of an integrated treatment model – psychosocial needs assessment	Medical oncology	Patients, cancer physician, oncology nurses, lab technicians admin staff	The integrated treatment model was driven by collaborative healthcare guidelines and a mission statement: The mission statement was: 1) to reduce suffering created by the effects of cancer on patients and loved ones; 2) to work with patients and their families to diminish stress and channel all available resources toward health, healing, and quality of living; 3) to provide ongoing support to staff and physicians; 4) to strengthen and support the physician/ patient relationship, and 5) to relieve staff and physicians of lengthy and/or complex psychosocial patient interventions. Proximity, accessibility and availability of the therapist enabled frequent team communication, a whole person approach, assessment of patient and family complex needs, resources, goals (lifestyle, faith, sexuality, grief). It supported crisis prevention and management. Outcomes were collective action, team shared learning, team satisfaction with care delivery, patient reduction in suffering, improved quality of life, and increased hope and perceived agency for patient and family.	1
Teno & Connor (2009) [53]	U.S.	Non-research Evidence-based commentary using a patient case	Hospice & palliative care	Physician, clinical nurse specialist, social worker, chaplain	Patient values, expectations, choice and cultural tradition, and patient dignity must be respected. The case exemplifies attendance to the patient physical and emotional comfort, evidence-based practice, shared decision making, family information needs. Team crisis care was available. Bereavement care was available before and after death. Compassionate care was coordinated across care settings and facilitated by IP referrals, transitions in care, and participation in important family events. An individualized, holistic care plan addressed the person’s priorities and contributed to perceived hope.	N/A
Thompson (2013) [59]	U.K.	Non-research Reflection	Schwartz Rounds in a UK hospital		Rounds require a supportive environment, **o**pen discussion between equals, time and facilities, Any member of staff, from porters to executive directors, can attend and participate. Senior clinicians acknowledged the complexity of the case and appreciated the team challenges. Positive outcomes were: improved communication, team members reminded of their value and why they entered a caring profession, emotional support from colleagues, catharsis, learning and understanding roles and challenges of others, celebrating achievements, led to **i**mproved patient experience	N/A
Wentlandt et al. (2016) [54]	Canada	Qualitative Interviews, focus groups	Hospital palliative care units	Physician, nurse, social work, PCU manager, chaplain, OT, Volunteer	Connection with patients and family members goes beyond just doing the job. A sense of community is appreciated by family. A quick response by the team was seen as “giving reassurance, pulling out all the stops and going the extra mile” to support a family’s wellbeing. Patients and caregivers expressed satisfaction with care that was seen to be “engaging”, sometimes humorous, and “genuine”.	(3)
Williams et al. (2008) [55]	Canada	Non-research Literature review	Perinatal /neonatal	Multidisciplinary team but specific reference to physician, social workers and nurses.	Families value speaking during meetings, continuity of the care team, timely communication, sharing sorrow, and being informed of changes in the care plan. Parents want to feel supported regardless of their decisions. Every team should exercise compassionate, individually tailored, and non-judgmental care including respectful treatment of the body. Respect can also be given through verbal and emotional support of family and co-workers. Shared decision-making should be viewed as a multidisciplinary process. The team should help parents feel that the right decision has been made. Compassionate care demonstrated through assistance with religious rites, funeral support, bereavement care. Interdisciplinary morbidity and mortality sessions or small group debriefings may help reduce the heavy emotional stress. Forming a connection with patients and family members and not “just do their job”	N/A

^a^ Appraisal grade scale: 1 = weak to 5 = strong based on criteria of Letts et al. [30] and Polit and Beck [31]

### Data presentation

A narrative summary of the synthesized findings with exemplar data sources is also consistent with Whittemore and Knafl’s integrative review method [26]. It is presented in the results section.

The overarching categories and sub-categories that reflect key indicators (structure, process, outcomes) of CCC at the individual, team, and organizational levels are displayed in Table 3.

**Table 3:** Data reduction and CCC operational framework

CCC Indicators	Individual Patient / Family / Provider	Interprofessional (IP) Team	Organization
Structures (attributes)	Overarching Structures: Patient and Family-Centered Care Overarching Values: Empathy, Sharing, Respect, and Partnership		
	Patient-Family Values & Expectations	Values	Culture
	• Commitment • Dignity • Supportive care	• Commitment • Authenticity • Holism	• Shared mission and vision • Leaders and champions • Inclusivity
	- Continuous - Non-judgmental	Skills	Policies
		• Relational • Leadership and advocacy • Reflection and self-awareness	• Support for IP patient-centered care
	Provider Needs & Expectations		Resources
	• Commitment • Support • Education		• Human (professional and non-professional) • Compassionate spaces • Time
		Resources	
		• Shared IP space • Time	
Processes (tools / mechanisms)	Overarching Processes: Communication, Shared decision-making, and Goal setting		
		Formal	Strategic planning
	• Symptom management • Spiritual care • Transitional care • Advance care planning • Bereavement care	• Care rounds and case conferences • Referrals and consultations • Transitional care • Advance care planning • Bereavement rounds • Schwartz Rounds	• To achieve priorities and goals
			Policy and program development
			• To support formal processes and pilot projects
		Informal	
		• Impromptu communication (hallway, telephone)	
Outcomes	Overarching Outcomes: Development and Satisfaction		
	Patient- Family Development and Satisfaction	Knowledge	Organizational Development
	• Self-care • Coping • Holistic care • Dignity and “being known” • Patient-provider relationships	• Complex end-of-life care • IP team roles and contributions	• Innovative programs and partnerships • Policies and processes
		Behavior	Organizational Satisfaction
		• IP communication • Collective purpose • Coping • Reflective practice	• Reduced provider burnout and compassion fatigue
	Provider Development and Satisfaction		
	• Patient-family goal achievement • Self-compassion • Self-care		
		Satisfaction	
		• Role fulfillment • Teamwork	

## Results

### Overarching findings

Based on data from 19 of 25 articles, our analysis revealed *‘patient and family centeredness’* as the primary structure for CCC across the individual, team, and organizational levels [37–55]. Overarching structural values were: a) empathy [39, 43, 46, 49, 54, 56–58], b) sharing [40, 46–48, 52, 53, 55, 56, 59], c) respect [37, 42, 44, 46, 47, 53, 55, 56, 59], and; d) partnership [37, 40, 42, 44, 46, 47, 49, 52–54, 59, 60]. Further to these findings, empathy, sharing, respect, and partnership are values that must be structurally present for CCC to evolve. In the literature sample, the act of co-suffering, or suffering alongside a patient and family, is demonstrated through compassionate presencing [48, 49, 57], as well as recognizing and acting on the presence of patient-family suffering [39, 43, 46, 54, 56–58].

“*You need to know that the people caring for you, whether they can or can’t help you with your disease, honor you for who you are and care about you”* [43].

Sharing is manifested when patients, families, and caregivers relate care concerns and preferences [52, 55, 56], learning needs [52], decisions [47, 53, 55], and care experiences [46, 47, 59]. Respect involves careful attention to the patient’s physical and bodily needs [55, 56], patient and family wishes [44], and verbal and emotional support for patient and family members [55, 56], as well as team members [37, 42, 46, 47, 59]. Finally, partnership involves forging formal and informal connections between patient, family, the team, organization, and external agencies or resources [37, 40, 42–44, 46–49, 52–54, 59, 60]. Partnerships among patients, families, and providers involve a shared journey [49, 59] that is not agenda driven and transcends sectors and settings [37].

### Structures

#### Individual structures

Patients and families value, need, and expect holistic and continuous care across the continuum [38, 39, 41, 49, 52, 55] that is supportive, non-judgemental and equitable [51–55].

*“Family member: I never felt like we weren’t part of your team… You always validated our thoughts and feelings... that’s so important to hear because we have to live with that [decision]”* [44].

Promoting and protecting dignity emerged as another important element of supportive care among patients and families [39, 43, 53, 56].

*“You [the patient] need to know that the people caring for you, whether they can or can’t help you with your disease, honor you for who you are”* [43].

With regard to providers, the analysis revealed personal and professional commitment [38, 42, 47, 58] as a prominent structure.

*“If I don’t do it from the heart, then the care isn’t good…I really don’t know what it is like to die*” [58].

Two additional provider structures that promote provider engagement in CCC include professional support [40, 42–44, 46, 47, 51, 52, 55, 59, 61] and education [39, 40, 51, 58, 61]. Education can be formal or informal, with patients and families sometimes serving as teachers:

*“Sometimes it’s learning from the family. Sometimes we’re not the expert. ‘You know your mother. You understand your culture…Help us…so that it’s meaningful for all of us’* [39].

#### Team structures

Attributes of CCC at the team level include shared values, skills, and resources. Teams must value authentic relationships [47, 50, 52, 56], a shared team commitment [42, 47, 56, 60], and a holistic approach that supports bio-psychosocial-spiritual care [40, 41, 44, 49, 58].

*“When a resident dies and they leave the home…the staff will line the corridors to say cheerio to them and that includes domestic staff, kitchen staff, everyone…I always go with the undertakers because I want to make sure that the person I’m looking after is still being looked after”* [56].

Skills at the team level are relational [49, 52, 54], and involve active listening [48, 49], leadership [50, 51, 56, 61], advocacy [51, 56], reflection and self-awareness [39, 43, 46–48, 59–61]. According to our analysis, human resources and time are key structural indicators at the team level. In particular, the literature sample supports an IP team approach in delivering CCC [37, 39, 40, 43–52, 54, 55, 58, 59]. Time and shared spaces for planning, sharing, and debriefing are essential to support CCC among teams [46, 47, 51, 52, 55, 59, 61].

*“…time needs to be allocated for this initiative to work, and it needs to be integrated into staff professional development as opposed to being a forum that can be attended only if staff have spare time*” [59].

#### Organizational structures

Nine articles revealed structural indicators within organizations that support CCC [37, 40, 50, 52, 54, 56, 58, 59, 61]. Three of those articles emphasized how a shared mission and vision for CCC can be influential in driving organizational programs and activities [51, 52, 61]. The organizational culture should be inclusive [56, 59] of all staff “from porters to executive directors” [59]. Leadership is essential for championing and supporting the planning [56, 61], and policies that promote IP patient and family-centred care may support CCC integration [40, 50, 56].

*“Organizational policies should promote and support spiritual compassionate care at the bedside, in the boardroom, and in staff relations”* [50].

Finally, adequate organizational resources are required for patient and family programs [37, 40, 52], IP staffing and support across the institution [40, 52, 59, 61], and compassionate spaces for patients and families [54, 58] as well as staff [56, 60].

*“We try to create a home-like rather than an institutional environment…. When everything is right, we’re sending a message that we do care…”* [58].

### Processes

Three overarching processes emerged at the individual, team, and organizational levels. These are: 1) communication [40, 43, 45, 46, 49, 52, 55, 56, 58, 61], 2) shared decision-making [39, 44, 45, 47, 53, 55] and 3) goal setting [37, 43, 44, 46–49, 51, 52, 56, 58, 60]. Not only do these processes bridge all levels, our analysis suggests that they may enable several CCC sub-processes among patients, families, teams, and organizations.

#### Individual and team processes

The sub-processes associated with CCC were similar across the individual and team levels: pain and symptom management [38, 41, 43, 44, 57], care rounds [41, 44, 58], case conferences, consultations and referrals [37, 43, 51, 53], spiritual care [38, 39, 43, 44, 49, 50, 52, 57, 58] advance care planning [52, 56], transitional care [47, 53, 55], and bereavement care [39, 46, 47, 53, 55]. Schwartz Center Rounds (SCR) emerged as an exemplar formal process through which CCC may be developed and sustained:

*“The more formal venues, such as the rounds or the chemo meetings, are not just meetings where we talk about what therapy someone’s on, they become, “Oh my God. She is 38. She has two kids and she has cancer”* [60].

SCR provides a venue for sharing the emotional work of caring with other carers [43–48, 60, 61], and this sharing can support CCC.

*“For the responsible and empathic practice of medicine, health-care providers have to engage in the routine process of reflecting, self-monitoring, processing emotion, and coping with its effects; tasks that are quite challenging without support. We have found the Rounds help to provide that support”* [57].

#### Organizational processes

Our analysis revealed three sub-processes in organizations that support CCC: 1) strategic planning [39, 51, 61], 2) policy development [37, 46, 56, 61], and 3) program development and evaluation 39, 40, 44, 49, 51, 52, 58, 61]. For example, palliative and end-of-life expertise was integrated in a U.S. pediatric hospital through strategic planning and development of a Compassionate Care Network [51]. In several instances, development of programs began with institutional pilot projects, such as the 3 Wishes Project [39], an integrated psychosocial treatment team [52], grief programming [51], and memorial services [40, 58]. These organizational processes may support the achievement of outcomes that are reported in the next section.

### Outcomes

Satisfaction and development emerged as the two overarching outcomes across all three levels. Knowledge and behavioral development occurred across the individual and team levels, whereas satisfaction emerged as a prominent outcome among patients, families, teams, and organizations.

Our analysis suggests that indicators of development and satisfaction may be evidenced by integrating the structures and formal processes that are described in the preceding results, however empirical study is required.

#### Individual outcomes

Indicators of patient-family knowledge and behaviour development include engagement in self-care [43, 61], enhanced patient-family coping [43, 44, 48, 52, 61], reduced fear and depression [48, 52, 60], and improved quality of life [41]. Patient satisfaction is reflected through “being known” [39, 43] holistically [39, 43, 49] by others on the care team. Finally, when compassion and collaboration are integrated in end-of-life care, patient and families report satisfaction with overall care delivery [54] and provider relationships [43, 44, 46, 52, 54].

*“This service is very important because of the intensity by which fear, love, anger, grief, stress, and loss overtake you. By giving compassion and tools to cope, patients and families are helped to love one another and stay connected. This is vital to making the process a healing one”* [52].

Provider satisfaction is associated with the achievement of patient end-of-life care goals, [43, 44, 46, 48, 49, 56, 58, 60], including spiritual peace [49, 60], pain and symptom management [43, 44, 48], and the provision of patient-family support across the continuum of care through bereavement [46, 58].

*“Both cure and healing fall within the responsibility of the health care profession. I think doctors and nurses offer the most powerful kind of healing possible when they really care about someone. You weren’t just a pro doing what you had to do. You went beyond being technically competent”* [46].

Finally, the ability to engage in self-care and self-compassion are additional prominent indicators of provider satisfaction [42, 56, 60, 61].

*“One must acknowledge the losses, accept the pain, strive to move beyond the grief, and then be willing to embrace new relationships guaranteed to include more loss”* [38].

#### Team outcomes

Knowledge development, behavioral development, and team satisfaction are the main team outcomes. Quality indicators of team knowledge development include expertise in managing complex end-of-life care [42–44, 51], including pain management [43, 44, 47, 51], and ethical decision-making [44]. Team behavioral development outcome indicators are: effective IP communication [42, 45, 51, 60], a collective purpose [39, 42, 47, 49, 60], strengthened team relationships [39, 43, 47, 49, 50, 60], and enhanced team coping 39, 47, 60, 61].

*“The synergy between all those interacting with the patient enhances the overall care and wellbeing of the patient. But it also makes the work of each individual healthcare professional richer in that the contribution of each healthcare member to the treatment plan potentiates each individual contribution. The wholeness is more than the sum of its parts”* [49].

According to our analysis, team satisfaction is evidenced through role fulfillment [44, 45, 48, 51, 56] and positive teamwork experiences associated with collectively achieving the patient-family goals of care [43–45, 51, 53].

*“It’s really important to bring someone out of the world. I think it’s a real privilege to do it. After they have passed away – changing them, laying them out and everything, putting the flowers on and seeing their family’s reactions when they see them like that – it makes you feel really proud of what you do”* [56].

#### Organizational outcomes

The findings suggest two main organizational outcomes, the first of which is organizational development. It can take the form of innovative programs, partnerships, and patient-centered changes in policy and practice [37, 40, 44, 46, 50, 51, 56, 61]. Examples of quality indicators include: evidence-based pain management protocols [44], institutional advance care planning procedures [56], spiritual care programming [50, 58], integrated acute care and community palliative teams [37, 51, 61], staff education and development [40, 51], Schwartz Rounds [60, 61], bereavement rounds [46, 51], and family bereavement care [39, 46, 51, 58].

*“Findings indicate high levels of engagement and intentionality about building community…equally important was the benefit of interdisciplinary exchange and understanding. Participants reported that the sessions increased their capacity to provide palliative care and integrate it into care on the units where they practiced. Participants in each [bereavement care]session identified specific new learning that would influence their clinical practice*” [51].

Secondly, indicators of organizational satisfaction that include reduced healthcare provider burnout and compassion fatigue emerged from several articles [42, 51, 56, 60, 61].

“The thing that keeps you going, even in the middle of a busy, frustrating day is when you can’t help all of the patients, is being able to connect with people. That is the only thing that keeps me coming back every day [clinical nurse practitioner]…I’ve been here…for about four years and have seen incredible changes. I’ve been thinking about how you survive in a place like this that keeps growing and growing and getting busier and busier every year…The goal for the day can be that you’ll connect someone…I hear the positive perceptions that patients have of their care providers…The regular newsletter “Hotline” occasionally publishes encouraging letters from patients. Reading these makes you feel really good because they identify the people that the patient had come into contact with [social worker]” [60]*.*

## Discussion

This integrative review was motivated by our shared practice experiences, and the voices of researchers, clinicians, and educators who advocate CCC as an essential component of healthcare quality [3, 62, 63]. To that direction, our work builds on the CCC Model and Framework [3] to promote operationalization of CCC in a way that is meaningful and measurable for patients and families who receive end-of-life care, as well as teams and organizations who provide end-of-life care.

To achieve this purpose, our integrative review process entailed an analysis and synthesis of the published literature related to CCC and end-of-life care over the last twenty years (1996 to 2016). Among the sample of 25 articles, less than one-third were published in the last five years. This finding indicates that CCC is an emerging field that has yet to receive the necessary attention by the scientific community, despite international calls for more compassionate care [3, 5, 63, 64]. The country of origin for the overwhelming majority of articles was the US (*n* = 17), followed by four Canadian articles, three from the UK, and one from Iran. This is not surprising as similar literature sample characteristics were reported in a recent scoping review of compassion [6] and a palliative care meta-analysis [65].

Patient and family-centered care was a dominant finding across the literature sample, and as such, we emphasize it as an overarching structure and key quality indicator of CCC. Patient and family-centered care is defined as “working *‘with’* patients and families, rather than just doing ‘*to’* or ‘*for’* them”, and it should take place in all settings and across all care levels [66]. At end-of-life, patients must be at the core of all end-of-life care processes, and families recognized as care team members, and not merely “visitors” [38]. Achieving a patient and family-centered care delivery model requires an extreme culture shift from a historic provider-driven model to one that involves patients and families in quality of care initiatives. This culture shift from passive, trusting and compliant patients, to engaged and empowered team members requires acquisition of a specific set of patient-centered care competencies [67]. However, according to critical social theory, integration of patient-centered care into health care organizations is frequently hindered by the inherent knowledge and power of healthcare providers [68]. According to our analysis, it also requires a sharing of values among patients, providers, teams, and organizations [37, 39, 40, 42–44, 46–49, 52–60].

The overarching structural values of empathy, sharing, respect, and partnership emerged across all ages of patients (infants through old age), and in acute care settings (NICU, ICU), tertiary care (hospice, palliative), and long-term homes/continuing care facilities. The same values were also revealed within North American, UK, and Iranian contexts. These findings are again not surprising, as the quality of patient-family and care provider relationships is fundamental to the social mission of hospice and palliative care [53, 58, 69, 70]. The structural values identified in this review are also reflected in the IPFCC’s four core concepts of patient and family-centered care, namely: respect, information sharing, participation and collaboration [66]. Empathy, although lacking in IPFCC’s concepts, is commonly accepted as a value in hospice and palliative care [71]. It is also often used as a synonym for compassion, although conceptually different [72]. Compassion extends empathy beyond merely understanding and acknowledging another’s experience, to include actions that are motivated by love and acts of kindness [72].

Communication, shared decision-making, and goal setting are three overarching processes that can support CCC. Acknowledging the abilities of other team members, as well as their contributions, is of great importance to engaging these processes [42]. In end-of-life care, the IP team includes the patient and his or her family, physicians, nurses, social workers, and the many professionals and non-professional volunteers who “cocoon” the dying patient [42]. The inclusion of patients and families expands previously accepted definitions of IP collaboration that only included professional caregivers [73]. Collaborating with, valuing, seeking, and offering support to this extended IP team are all important attributes of CCC. The team seeks communication at all levels, and understanding of how the environment influences care [58] to integrate meaningful processes, such as honoring dying patient wishes, humanizing the environment, offering tributes, facilitating family reconnections, rituals and observances, and “paying it forward” [39]. More formal IP team processes include care conferences, rounds, advance care planning, and are listed as quality indicators of CCC. A significant finding from this review is the value of formalized team rounds, and their impact on provider self-care and emotional regulation [43–48], with SCR being an exemplar case of CCC.

Eight articles reported narrative summaries of SCR with great richness and depth in dialogue [33–48, 60, 61], and poignant descriptions of each healthcare provider’s unique perspectives and contributions to the IP care plan. SCR are multidisciplinary forums where HCPs come together to discuss and process emotionally and ethically complex care issues [74]. In these rounds, reflection on the emotional aspect of care strengthens a provider’s ability to deal with similar situations in the future, providing support towards empathic practice [61]. For palliative and end-of-life care, SCR provide an ideal milieu for promoting compassion and IP teamwork among attendees. According to Manning and colleagues, SCR are very well received by healthcare professionals [75]. Moore and Phillips report improved attendee insights into psychosocial aspects of patient care, teamwork, and less clinical isolation [61]. Issues raised by staff during SCR center around three concerns: (1) staff uneasiness with a patient’s decision for continuing or discontinuing a therapeutic regime, (2) verbalizing the need to say goodbye to a patient at end-of-life, and (3) going through the emotions elicited by the death of a patient with whom a provider identified and bonded [45]. Unexpected positive outcomes include patient-centered changes in institutional policy or practice, greater use of palliative care teams/enhanced palliative care services, and discussion among staff about advanced illness and palliative care issues [61]. Implementing SCR requires human resources, advanced planning, and commitment by institutional administration [59, 61].

Development and satisfaction emerged as overarching outcomes at the individual, team and organization levels. Outcomes such as self-care, dignity, self-compassion, holistic care provision, therapeutic patient-provider relationships, and goal achievement [43, 44, 48, 49, 52, 56, 61] are important indicators for evaluating quality care among patients, families, as well as professional and non-professional caregivers. Examples include “giving voice to the family”, and promoting family involvement in the caring process [39]. Several of these outcomes can be measured to evaluate quality. For example, the Patient Dignity Inventory is a reliable and valid measure for measuring dignity-related distress at end-of-life [76]. Walker and colleagues recently developed and tested a scale to measure patient perspectives of holistic and integrated care [77]. The McGill Quality of Life Questionnaire is widely used among individuals with advanced disease and at end-of-life [78].

Key indicators of team development include interdependency and synergy [39, 47, 49, 52, 60, 61]. This finding is not surprising as these concepts are attributes of IP collaboration [73]. According to the American Academy of Pediatrics guidelines, children’s hospitals should have dedicated interdisciplinary pediatric palliative care and hospice care (PPC-PHC) teams [37]. These teams provide integrated multimodal care (cure seeking, life-prolonging, comfort-enhancing, quality-enriching), facilitate clear and compassionate discussions, and support families and staff beyond the end-of-life period [37]. A recent systematic review by Mulvale and colleagues reveals that interrelated ‘gears’ at the macro, meso, micro and individual levels are critical considerations for IP collaboration [79]. Although focused on primary care, Mulvale’s findings are similar to those of this review in that dedicating human resources, setting a common vision, attending to formal and social processes, and valuing the contributions of team members are highly recommended actions. Continuous improvement activities, such as quality audits and regularly scheduled team meetings, are equally important to understanding how policy and organizational contexts affect the ability of teams to collaborate effectively [79]. According to the gears model, collaboration should extend beyond the team itself to include policy-makers, organizational leaders, team leaders and individual professionals [79].

### Strategies to enable CCC

Our findings draw attention to environmental factors at all three levels that can enable or hinder CCC, and are congruent with the recently published compassionate care flow model by Tierney et al. [80]. This study examines how compassionate care is delivered to patients with type 2 diabetes within a range of healthcare settings [80]. This model demonstrates that mere intention to providing compassionate care is not enough. Rather, working within an environment that supports compassionate practice is perhaps more important. The flow of compassionate care can be enhanced by defenders (i.e. empathizing with patient, supportive colleagues, professional autonomy, faith, controlling own emotions) and/or depleted by drainers (i.e. competing agendas, time and resource limitations, negative emotions). Compassionate care is learned within the work environment, and shaped by the influence of colleagues, patients and organizational demands and expectations [80]. Nevertheless, the extent to which an organization can modify provider behavior, and enhance CCC performance is under debate [9]. Regardless, our findings complement previous research which suggests that organizations and systems can enable rather than impede compassionate, high quality healthcare [1, 2]. Main enablers include: 1) resource allocation and policy setting focusing on the needs of patients/families and caregivers (professionals and non-professionals), 2) valuing and recognizing compassionate caregivers and organizations, 3) supporting providers to manage the emotional stress of caring, and to diminish personal or moral distress, and burnout, 4) forming partnerships with patients and families, 5) educating providers, patients, and families about the attributes and benefits of CCC, and 6) developing flexible QI processes to implement and continuously improve compassionate care [80].

When conducting the analysis, *commitment* and *support* were coded with high frequency at the individual and team levels. The importance of ongoing support from relatives, friends, and the team as resources, described as “circles of strength” and having “a safety net” emerged as exemplars [43, 44]. Nevertheless, there were wide variations in how these indicators were reported. Given the subjectivity, these indicators need to be interpreted from a clinical perspective. That is, when discussing goals of care, a meaningful ongoing assessment should occur. The following practical and powerful question for patients and families should be routinely asked: *“How can I and/or the team demonstrate commitment to you and how can I / we support you in your journey?”* Our analysis also suggests that individual practitioners and teams require ongoing organizational support; the attributes and processes of support should be systematically assessed and implemented by institutional leaders.

Several strategies to promote and engage individuals, teams, and organizations in CCC were discussed in the sample articles. Among them, the Comprehensive Pediatric Bereavement Program is characterized by a team approach, recognition of cultural differences, integration of family into care of the dying, support groups, resource lists and information, remembrance ceremonies, continued contact with family, staff education and development, program evaluation and feedback [40]. The most documented strategy, SCR, is developed and sponsored by the Schwartz Center for Compassionate Healthcare [74]. The Schwartz Center supports individual organizations to implement SCR through providing educating and training programs in compassionate care. The Schwartz Center’s *“Compassion in Action Webinar Series”* teaches participants how to sustain compassion and collaboration in healthcare while sustaining one’s well-being [74]. Presenters teach some of the concepts and skills that are essential components of the CCC model in ways that are meaningful to patients, families and providers. For example, the 2017 webinar series includes CCC training at the organization and systems level.

Because the required skills to deploy empathy and compassion are not routinely taught nor systematically assessed and evaluated across the continuum of learning and practice [3], targeted measures and policies that reinforce humanistic values, such as kindness and compassion, are important in healthcare institutions and in healthcare education [5, 81]. Recently, a UK educational institution introduced SCR in undergraduate medical education [81]. Medical students perceived SCR to support their self-reflection, insight and emotional processing [81]. Challenges include training, cost, optimal timing, and participation [59, 61, 74, 81].

At the organizational level, the use of indicators allows for ongoing monitoring of health care quality, setting the basis for quality improvement (QI) and prioritization in the healthcare system [23]. Rushton and team evaluated four QI initiatives at a U.S. Children’s Hospital that included: 1) the establishment of a Compassionate Care Network that spanned all units of the institution, 2) institutional palliative care rounds, 3) patient care conferences, and 4) bereavement debriefing [51]. The above QI initiatives can enable CCC, however both top-down and bottom-up organizational commitment and support must be enacted [82]. As highlighted in the IMPACT study, the use of quality indicators to drive improvements in palliative care settings is determined by the organization’s orientation towards continuous improvement. Furthermore, sustainability is determined by the perceived value of the QI package which can differ across settings (i.e. specialist palliative care vs. generalist care). Finally, ‘top-down’ engagement approaches were reported to be less effective [82].

### Implications

We assert palliative and end-of-life care as the ‘gold standard’ for operationalizing CCC. Given that palliative care should begin once a life-limiting condition is diagnosed [19, 70], the majority of patients and families who access healthcare can benefit from CCC. The findings of this review can be applied by institutions and systems implementing and maintaining a culture of CCC as part of QI, accreditation and/or magnet status projects.

**Fig. 3:**
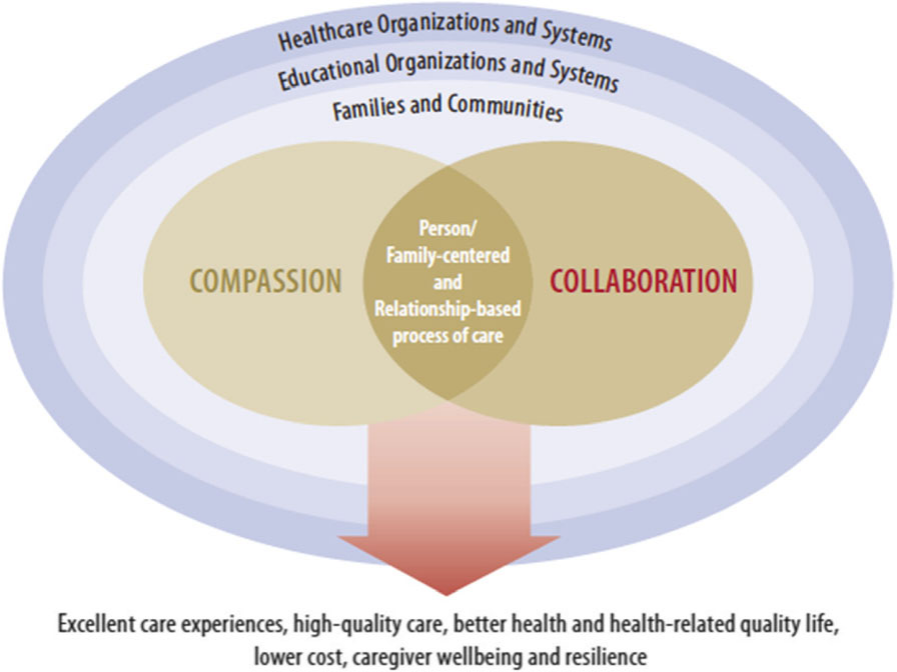
Context and outcomes of compassionate collaborative care

Our study validates the work of the Schwartz Center for Compassionate Healthcare and the Arnold P. Gold Foundation whose visual representation (Fig. 3) shows how person-family-centered care can be achieved when compassion and collaboration intersect and are supported within the family, community, education and healthcare systems [3]. In addition, our work contributes to the understanding of the quality indicators within each system, with exception of the community and educational systems. It indicates how the CCC approach can optimize patient-family and provider outcomes, such as satisfaction with care and satisfaction with providing care, respectively. Although our analysis did not reveal key indicators related to staff turnover, it is reasonable to hypothesize that reductions in burnout/compassion fatigue will positively affect provider and staff retention [83].

As previously highlighted, the usefulness and applicability of the review’s findings outside of facility-based end-of-life care cannot be assured. The community requires in-depth exploration, with Compassionate Communities representing an opportunity for comprehensive understanding. ‘*Compassionate Communities’* or *‘Compassionate Cities’* are examples of CCC that are applied using a public health approach to comprehensively address end-of-life care at the community level [84]. Now expanding across the globe, *‘Compassionate Communities’* engage citizens to partner with HCPs and others to meet the holistic healthcare needs identified by patients and families [84]. As more of these communities evolve, evaluation of the fit of the quality indicators in the community setting will be interesting and may increase its utility.

Reliable and valid indicator measures will be required to measure quality outcomes of CCC. *‘The Schwartz Center Compassionate Care Scale’* is a new instrument that measures patients’ perceptions of compassionate care provided by hospital physicians [85]. It is unknown if the scale has been tested in end-of-life care settings, and the instrument does not provide a level of team engagement in CCC. With the growing debate on the ability to measure CCC, experts urge the inclusion of compassionate care elements in national surveys of patient experience using standardized protocol items [85]. Sinclair’s team is actively developing a patient-reported instrument to measure compassionate care, and this work will support the advancement of CCC within teams and organizations [86]. Although patient and family satisfaction with healthcare is a quality indicator valued by most organizations, policy and institutional decision-makers are primarily driven by economic and high quality clinical data. Future work is needed to evaluate the benefits of CCC on costs, efficiencies, staff turnover and retention. Evaluation will be challenged by the complexity of the concept. Randomized controlled studies remain the bedrock of evidence-based practice, and their application in evaluating complex interventions can be fraught with challenges [87]. Pragmatic trials and mixed methods studies may be more feasible to generate the strength of evidence needed to change practice and policy [88].

### Strengths & limitations

Our review adds to the existing body of knowledge and builds on the recent work and recommendations of several professional organizations and experts [3, 6, 7]. It overcomes the limitations identified by Gaertner et al. by providing an in-depth analysis of a complex phenomenon [89]. With regard to the review process, rigor was supported through a comprehensive search strategy, using explicit inclusion and exclusion criteria. Two authors independently reviewed each citation and abstract, and a database was developed ‘a priori’ to support the organization and sharing of data. The authors met and reviewed their assessments and decisions, and came to consensus for all discrepancies.

This review is limited by a largely US literature sample, and the focus was end-of-life care. Therefore, the findings cannot be generalized beyond this population. There were no studies that explicitly examined the quality indicators of CCC as a primary outcome. The overall quality of the literature sample was weak since over two thirds reflected position/consensus statements, case studies, literature reviews and SCR reports which could not be appraised. Inter-rater reliability for the abstract reviews was not calculated. The usefulness of the quality indicators outside of end-of-life care settings will be contingent on the organization’s structures and processes. Future studies and pilot implementation are required to further refine the key indicators.

## Conclusion

Compassionate collaborative care (CCC) is an emerging, complex concept. Although limited by a lack of strong empirical evidence, it is of growing importance for healthcare quality. This integrative review suggests that CCC is inextricably linked to the inherent values, needs and expectations of patients, families and healthcare providers. Communication, shared decision-making and goal setting comprise the overarching processes, while development and satisfaction are overarching outcomes. These findings may be applied to facilitate the assessment and evaluation of existing structures, processes, and outcomes at the patient-family, provider, team, and organizational levels, and guide the planning of team and organizational changes to achieve the essential quality indicators for CCC. Given the growing numbers of individuals who require quality end-of-life care [19, 84], this review provides a synthesis of the evidence for clinicians, administrators, and policy makers wishing to maximize the delivery of CCC in palliative and end-of-life care settings.

## Abbreviations

CCC: Compassionate collaborative care
CIHC: Canadian Interprofessional Health Collaborative
ICU: Intensive care unit
IP: Interprofessional
NICU: Neonatal intensive care unit
PPC-PHC: Pediatric palliative care and hospice care
QI: Quality improvement
SCR: Schwartz center rounds

## References

[1] Lown BA (2014). Seven guiding commitments: making the U.S. healthcare system more compassionate. J Pt Experience.

[2] Lown B (2015). Compassion is a necessity and an individual and collective responsibility. Int J Health Policy Manag..

[3] The Schwartz Center for Compassionate Healthcare and the Arnold P. Gold Foundation (2014). Advancing compassionate, person-and family-centered care through inter-professional education for collaborative practice. Recommendations from a conference on advancing compassionate, person-and family-centered care through Interprofessional education for collaborative practice.

[4] Department of Health (2015). The NHS constitution: the NHS belongs to us all.

[5] Fotaki M (2015). Why and how is compassion necessary to provide good healthcare. Int J Health Policy Manag.

[6] Sinclair S, Norris JM, McConnell SJ, Chochinov HM, Hack TF, Hagen NA, McClement S, Bouchal SR (2016). Compassion: a scoping review of the healthcare literature. BMC Palliat Care..

[7] Lown BA, Rosen J, Marttila J (2011). An agenda for improving compassionate care: a survey shows about half of patients say such care is missing. Health Aff.

[8] Schantz ML (2007). Compassion: a concept analysis. Nurs Forum.

[9] Cole-King A, Gilbert P (2011). Compassionate care: the theory and the reality. J Holistic Healthcare.

[10] Government of Canada. Healthy workplaces. https://www.canada.ca/en/health-canada/services/health-care-system/health-human-resources/strategy/healthy-workplaces.html. Accessed 20 Sept 2016.

[11] Canadian Interprofessional Health Collaborative (CIHC).A national interprofessional competency framework. https://www.cihc.ca/files/CIHC_IPCompetencies_Feb1210.pdf. Accessed 15 Sept 2017.

[12] World Health Organization (WHO). Department of Human Resources for Health. Framework for action on interprofessional education and collaborative practice. 2010. Ref #**:** WHO/HRH/HPN/10.3 http://www.who.int/hrh/resources/framework_action/en/. Accessed 26 June 2017.

[13] Barrett J, Curran V, Glynn L, Godwin M. CHSRF synthesis: Interprofessional collaboration and quality primary healthcare. Ottawa: Canadian Health Services Research Foundation. 2007. Available at: http://www.cfhi-fcass.ca/Migrated/PDF/SynthesisReport_E_rev4_FINAL.pdf. Accessed 19 July 2017.

[14] Bankston K, Glazer G. Legislative: Interprofessional collaboration: What’s taking so long? OJIN. 2013;19(1). doi:10.3912/OJIN.Vol18No01LegCol01. 26812197

[15] Pfaff K, Markaki A, Echlin J, Hamilton L. Collaborative practice revisited: Compassion as the missing antecedent. Sigma Theta Tau International 43rd Biennial Convention Program, Las Vegas, Nov. 7–11, 2015.

[16] Dame Cicely BMJ (2005). Saunders. Founder of the modern hospice movement. BMJ.

[17] Hanks G, Cherny N, Portenoy R, Kaasa S, Fallon M, Christakis N. Introduction to the fourth edition: Facing the challenges of continuity and change. In Oxford Textbook of Palliative Medicine (4th ed). Edited by Hanks G, Cherny N, Christakis N, Fallon M, Kaasa S, Portenoy R. New York: Oxford; 2010.

[18] Murray S, Sheikh A (2008). Care for all at the end of live. BMJ.

[19] World Health Organization (WHO). (2017). WHO definition of palliative care. http://www.who.int/cancer/palliative/definition/en/. Accessed 26 June 2017.

[20] Lionis C (2015). Why and how is compassion necessary to provide good healthcare? Comments from an academic physician. Int J of Health Policy Manag.

[21] The Arnold P. Gold Foundation. Gold Foundation Launches Triple C Initiative to Promote Compassionate, Collaborative Care. http://www.gold-foundation.org/newsroom/blog/gold-foundation-launches-triple-c-initiative-to-promote-compassionate-collaborative-care/. Accessed 26 June 2017.

[22] Schuster MA, Asch SM, McGlynn EA, Kerr EA, Hardy AM, Gifford DS (1997). Development of a quality of care measurement system for children and adolescents. Methodological considerations and comparisons with a system for adult women. Arch Pediatr Adolesc Med.

[23] Mainz J (2003). Defining and classifying clinical indicators for quality improvement. Int J Qual Health Care.

[24] Lloyd M, Carson A (2011). Making compassion count: equal recognition and authentic involvement in mental health care. Int J Consumer Stud.

[25] Van der Cingel M (2011). Compassion in care: a qualitative study of older people with a chronic disease and nurses. Nurs Ethics.

[26] Whittemore R, Knafl K (2005). The integrative review: updated methodology. J Adv Nurs.

[27] Donabedian A (1966). Evaluating the quality of medical care. Milbank Mem Fund Q.

[28] Donabedian A (1988). The quality of care. How can it be assessed?. J Am Med Assoc.

[29] Aromataris E, Fernandez R, Godfrey C, Holly C, Kahlil H, Tungpunkom P (2015). Summarizing systematic reviews: methodological development, conduct and reporting of an umbrella review approach. Int J Evid Based Healthc.

[30] Letts L, Wilkins S, Law M, Stewart D, Bosch J & Westmorland M. Critical review for qualitative studies (version 2.0); 2007. https://srs-mcmaster.ca/wp-content/uploads/2015/05/Guidelines-for-Critical-Review-Form-Qualitative-Studies.pdf. Accessed 20 July 2017.

[31] Polit D, Beck C (2004). Nursing research. Principles and methods.

[32] Cullum N, Ciliska D, Haynes RB, Marks S (2008). Evidence-based nursing: an introduction.

[33] Littlewood C, Chance-Larsen K, McLean SM (2010). Quality appraisal as a part of the systematic review: a review of current methods. Int J Physio and Rehab.

[34] Corbin JM, Strauss AL (2008). Basics of qualitative research: techniques and procedures for developing grounded theory.

[35] Miles MB, Huberman M (1994). Qualitative data analysis: an expanded sourcebook.

[36] Crabtree BF, Miller WL (1999). Doing qualitative research.

[37] American Academy of Pediatrics (2013). Pediatric palliative care and hospice care commitments, guidelines, and recommendations. Pediatrics.

[38] Borhani F, Hosseini S, Abbaszadeh A (2014). Commitment to care: a qualitative study of intensive care nurses' perspectives of end-of-life care in an Islamic context. Int Nsg Review.

[39] Cook D, Swinton M, Toledo F, Clarke F, Rose T, Hand-Breckenridge T (2015). Personalizing death in the intensive care unit: the 3 wishes project: a mixed-methods study. Ann Intern Med.

[40] Cox S (2004). Pediatric bereavement: supporting the family and each other. J Trauma Nsg.

[41] Hanson E, Cullihall K (1996). Palliative nursing care of a man with terminal cancer. Br J Nurs.

[42] Kehoe M (2006). Embodiment of hospice nurses. J Hospice Palliat Nsg.

[43] Knuti K, Wharton R, Wharton K, Chabner B, Lynch TJ, Penson R (2003). Schwartz center rounds. Living as a cancer surpriser: a doctor tells his story. Oncologist.

[44] Krakauer E, Penson R, Truog R, King L, Chabner B, Lynch TJ (2000). Schwartz center rounds. Sedation for intractable distress of a dying patient: acute palliative care and the principle of double effect. Oncologist.

[45] Lintz K, Penson R, Cassem N, Harmon D, Chabner B, Lynch TJ (1999). Schwartz center rounds. A staff dialogue on aggressive palliative treatment demanded by a terminally ill patient: psychosocial issues faced by patients, their families, and caregivers. Oncologist.

[46] Penson R, Green K, Chabner B, Lynch TJ (2002). Schwartz center rounds. When does the responsibility of our care end: bereavement. Oncologist.

[47] Penson R, Rauch P, McAfee S, Cashavelly B, Clair-Hayes K, Dahlin C (2002). Schwartz center rounds. Between parent and child: negotiating cancer treatment in adolescents. Oncologist.

[48] Penson R, Partridge R, Shah M, Giansiracusa D, Chabner B, Lynch TJ (2005). Schwartz centre rounds. Update: fear of death. Oncologist.

[49] Puchalski C, Lunsford B, Harris M, Miller R (2006). Interdisciplinary spiritual care for seriously ill and dying patients: a collaborative model. Cancer J.

[50] Puchalski CM, Vitillo R, Hull SK, Reller N (2014). Improving the spiritual dimension of whole person care: reaching national and international consensus. J Palliat Med.

[51] Rushton C, Reder E, Hall B, Comello K, Sellers D, Hutton N (2006). Interdisciplinary interventions to improve pediatric palliative care and reduce health care professional suffering. J Palliat Med.

[52] Schermer Sellers T (2000). A model of collaborative healthcare in outpatient medical oncology. Fam Syst Health.

[53] Teno J, Connor S (2009). Referring a patient and family to high-quality palliative care at the close of life. JAMA.

[54] Wentlandt K, Seccareccia D, Kevork N, Workentin K, Blacker S, Grossman D, Zimmermann C (2016). Quality of care and satisfaction with care on palliative care units. J Pain Symptom Manag.

[55] Williams C, Munson D, Zupancic J, Kirpalani H (2008). Supporting bereaved parents: practical steps in providing compassionate perinatal and neonatal end-of-life care. A north American perspective. Semin Fetal Neonatal Med.

[56] Addicott R (2011). Supporting care home residents at the end of life. Int J Palliat Nsg.

[57] Costello J (2001). Nursing older dying patients: findings from an ethnographic study of death and dying in elderly care wards. J Adv Nurs.

[58] Kayser-Jones J, Chan J, Kris A (2005). A model long-term care hospice unit: care, community, and compassion. Geriatric Nsg.

[59] Thompson A (2013). How Schwartz rounds can be used to combat compassion fatigue. Nurs Manag.

[60] Penson R, Dignan F, Canellos G, Picard C, Lynch TJ (2000). Schwartz center rounds. Burnout: caring for the caregivers. Oncologist.

[61] Moore C, Phillips J (2009). In these rounds, health-care professionals heal themselves. J Soc Work End-Of-Life Palliat Care.

[62] Paterson R (2011). Can we mandate compassion?. Hast Cent Rep.

[63] Francis R (2013). Report of the mid Staffordshire NHS foundation.

[64] World Health Organization (WHO) (2017). Seventieth world health assembly opens in Geneva.

[65] Kavalieratos D, Corbelli J, Zhang D, Dionne-Odom JN, Ernecoff NC, Hanmer J, Hoydich ZP, Ikejiani DZ, Klein-Fedyshin M, Zimmermann C, Morton SC, Arnold RM, Heller L, Schenker Y (2016). Association between palliative care and patient and caregiver outcomes: a systematic review and meta-analysis. JAMA.

[66] Institute for Patient and Family-Centred Care (n.d.) Patient and family-centred care. http://www.ipfcc.org/about/pfcc.html Accessed 13 July 2017.

[67] Bernabeo E, Holmboe ES (2013). Patients, providers, and systems need to acquire a specific set of competencies to achieve truly patient-centered care. Health Aff.

[68] Fredericks S, Lapum J, Schwind J, Beanlands H, Romaniuk D, McCay E (2012). Discussion of patient-centered care in health care organizations. Qual Manag Health Care.

[69] DiTullio M, MacDonald D (1999). The struggle for the soul of hospice: stress, coping, and change among hospice workers. Am Hosp Palliat Care.

[70] Doyle D, Woodruff R (2013). The IAHPC manual of palliative care.

[71] Doyle D (2004). The essence of palliative care: a personal perspective.

[72] Sinclair S, Beamer K, Hack TF, McClement S, Bouchal SR, Chochinov HM, Hagen NA (2017). Sympathy, empathy, and compassion: a grounded theory study of palliative care patients’ understandings, experiences, and preferences. Palliat Med.

[73] D’Amour D, Oandasan I (2004). Interprofessional education for collaborative patient-centred practice: an evolving framework.

[74] The Schwartz Center. Schwartz Center Rounds. 2016. http://www.theschwartzcenter.org/supporting-caregivers/schwartz-center-rounds/. Accessed 26 June 2017.

[75] Manning C, Acker M, Houseman L. Schwartz Center Rounds® evaluation report. Executive summary. Goodman Research Group. 2008. http://www.theschwartzcenter.org/media/PTXAAE65CHR5UU4.pdf. Accessed 17 July 2017

[76] Chochinov HM, Hassard T, McClement S (2008). The patient dignity inventory: a novel way of measuring dignity-related distress in palliative care. Pain Symptom Manag.

[77] Walker K, Stewart AL, Grumbach K (2016). Development of a survey instrument to measure patient experience of integrated care. BMC Health Serv Res.

[78] Cohen SR, Mount BM, Strobe MG (1995). The McGill quality of life questionnaire: a measure of quality of life for people with advanced disease. Palliat Med.

[79] Mulvale G, Embrett M, Razavi SD (2016). ‘Gearing up’ to improve interprofessional collaboration in primary care: a systematic review and conceptual framework. BMC Fam Practice.

[80] Tierney S, Seers K, Tutton E, Reeve J (2017). Enabling the flow of compassionate care: a grounded theory study. BMC Health Serv Res.

[81] Gishen F, Whitman S, Gill D, Barker R, Walker S (2016). Schwartz Centre rounds: a new initiative in the undergraduate curriculum—what do medical students think?. BMC Med Educ.

[82] Iliffe S, Davies N, Manthorpe J (2016). Improving palliative care in selected settings in England using quality indicators: a realist evaluation. BMC Palliat Care..

[83] Wood BD, Killion JB (2007). Burnout among healthcare professionals. Radiol Manage.

[84] Kellehear A (2005). Compassionate cities. Public health and end of life care.

[85] Lown BA, Muncer SJ, Chadwick R (2015). Can compassionate healthcare be measured? The Schwartz center compassionate care scale. Pat Educ Couns.

[86] B.C. Centre for Palliative Care. Research projects. http://www.bc-cpc.ca/cpc/research-projects/. Accessed 20 July 2017

[87] Coly A, Parry G. Evaluating complex health interventions: a guide to rigorous research designs. Washington: Academy Health; 2017. http://www.academyhealth.org/evaluationguide. Accessed 14 July 2017

[88] Zwarenstein M, Treweek S (2009). Making trials matter: pragmatic and explanatory trials and the problem of applicability BMC. Trials.

[89] Gaertner J, Siemens W, Daveson BA (2016). Of apples and oranges: lessons learned from the preparation of research protocols for systematic reviews exploring the effectiveness of specialist palliative care. BMC Palliat Care.

